# Preincision versus postincision frequent door openings during total joint arthroplasty

**DOI:** 10.1017/ash.2021.247

**Published:** 2022-01-12

**Authors:** Danielle N. Davis, Lexie K. Ross, Zihan Feng, Ryan Imber, Craig Hogan, Heather L. Young

**Affiliations:** 1 University of Colorado School of Medicine, Aurora, Colorado; 2 University of Colorado Health, Aurora, Colorado; 3 Denver Health, Denver, Colorado

Surgical site infections (SSIs) are a serious complication of total hip and total knee arthroplasty.^1^ Risk factors for developing SSIs can be considered in 4 categories: (1) patient-related factors, (2) surgical technique, (3) operating room environment,^2^ and (4) postoperative care. Of these factors, the operating room environment stands out as the factor that healthcare professionals have the most control over. Frequent operating room door openings are believed to disrupt laminar airflow^3^ and positive pressure.^4^ Several studies have implicated frequent door openings in the operating room with higher rates of airborne contamination^3,5^ and subsequently increased rates of SSIs.^6^

High rates of door openings during total hip and total knee arthroplasty have been previously reported in the literature.^1,5,7,8^ However, the difference in door openings between the preincision period and the postincision period has not been clearly defined. This factor is significant; previous studies have shown an increase in airborne contamination during the preincision period compared to the postincision period.^3^ Therefore, we sought to understand the reasons for door openings in the preincision and postincision periods to provide insight on how to best develop interventions for these 2 periods.

## Methods

This study was cross-sectional and observational in design. Data were recorded at 3 large academic institutions between June 2019 and August 2020. Total hip and knee arthroplasty procedures were included. Revision procedures met exclusion criteria. Observations were made by 4 observers who all underwent identical training and used a standardized data collection form. The number of door openings was recorded as well as the reason for the door openings and the period in which the door was opened. Additionally, distractions associated with door openings were recorded and rated according to severity using a scale adopted from Healey et al.^9^

The preincision period was defined as the time between the opening of the sterile instrument tray to the first incision. The postincision period was defined as the time between the first incision and the application of the bandage. This study met the classification for “not human subject research” by our institutional review board. Data were analyzed using the Wilcoxon 2-sample median test.

## Results

In this study, we observed 25 preincision sessions and 26 postincision sessions. Among them, 11 were total knee arthroplasties and 15 were total hip arthroplasties. The preincision period was a median of 56 minutes (IQR, 49–63). The median duration of surgery (postincision period) was 81 minutes (IQR, 67–91). Overall, we recorded 0.56 (IQR, 0.40–0.70) door openings per minute in the preincision period and 0.34 (IQR, 0.26–0.45) door openings per minute in the postincision period. We detected a significant difference between these 2 periods (*P* = .0036). The results were uniform across all 3 sites.

The following reasons were given for door openings in the preincision period, including the median number per case: 8 (25%) nurses obtaining supplies; 7 (20%) surgical team (ie attending physicians, residents, and medical students) entering and leaving the OR to check on the progress of the surgical preparation; and 7 (19%) other (eg, medication deliveries and nursing students entering and leaving) (Table 1). The following reasons were given for door openings in the postincision period, including the median number per case: 6 (18%) nurses obtaining supplies, 6 (18%) vendor getting supplies; 8 (17%) other (eg, radiology techs entering and leaving the operating room for radiograph-dependent cases or case-related questions) (Table 1).

**Table 1.** Reasons for Preincision and Postincision Door Openings

Variable	Total Staff Break	Nurse Supplies	Vendor Supplies	Surgical Team	Hallway Door	Other
**Preincision**						
Median no. per case	0	8	2	7	3	7
% of total door openings	0	25.40	5.43	20.53	11.98	19.74
**Postincision**						
Median no. per case	4	6	6	1	0	8
% of total door openings	12.50	18.60	18.75	3.7	0	17.14

Furthermore, 36% of door openings with a subsequent question or discussion regarding surgical equipment were rated as severe distractions (7–9 on the 9-point distraction severity scale adopted from Healey et al^9^). Also, 70% of door opening distractions associated with case irrelevant talk were rated as a mild distractions (1–3 on the 9-point scale). In addition, 97% of door openings that did not result in a subsequent conversation were rated as mild distractions (1–3 on the 9-point scale).

## Discussion

Our results are similar, although somewhat lower than previously reported door openings, such as Bedard et al,^8^ who reported a rate of 0.84 door openings per minute in the preincision period and a rate of 0.54 door openings per minute in the postincision period. Based on our findings, it is unlikely that the surgical team is significantly distracted by the high rates of door openings. However, 36% of door openings associated with a question or conversation regarding surgical equipment were rated as a severe distractions and may contribute to surgical error and increased risk of SSI.

Although the literature on the effect of door openings during the postincision period is grwoing, little is known about the impact of door openings during the preincision period.^1,8^ Given the previously reported significant increase in airborne contamination during the preincision period^3^ and the high rate of preincision door openings, it is reasonable to hypothesize that door openings^3,5,10^ may affect the sterility of the instrument tray. We found a significant difference in the reasons for door opening between the preincision and postincision periods, which signifies that their roles in the increased rates of SSI are likely distinct and that they should be investigated separately.

Nurse and vendor supplies constituted a considerable number of preincision door openings (25% and 5%, respectively) and postincision door openings (18% and 18%, respectively). A promising intervention to address these door openings would be the implementation of a checklist to ensure the presence of all necessary supplies prior to the preincision period. Further research is needed to understand the effect of door openings in the preincision and postincision period as well as to discover an effective and sustainable door-opening intervention.
